# The impact of COVID-19 first wave national lockdowns on perinatal outcomes: a rapid review and meta-analysis

**DOI:** 10.1186/s12884-021-04156-y

**Published:** 2021-10-06

**Authors:** Christine Vaccaro, Farida Mahmoud, Laila Aboulatta, Basma Aloud, Sherif Eltonsy

**Affiliations:** 1 College of Pharmacy, Rady Faculty of Health Sciences, University of Manitoba, Winnipeg, Canada; 2 Faculty of Pharmacy, Universite de Montreal, Montréal, Canada; 3 The Children’s Hospital Research Institute of Manitoba, Winnipeg, Canada

**Keywords:** Low birth weight, Stillbirth, Preterm birth, COVID-19, Lockdown

## Abstract

**Background:**

Since the emergence of COVID-19, preventative public health measures, including lockdown strategies, were declared in most countries to control viral transmission. Recent studies and anecdotes have reported changes in the prevalence of perinatal outcomes during national COVID-19lockdowns.The objective of this rapid review was to evaluate the impact of COVID-19 lockdowns on the incidence of low birth weight (LBW), preterm birth (PTB), and stillbirth.

**Methods:**

Two reviewers searched EMBASE, CORD-19, LitCovid (PubMed), WHO Global research on corona virus disease (COVID-19), and MedRxiv for studies published in English from the first reports on COVID-19 until 17 July 2021. Perinatal outcomes of interest included LBW (< 2500 g), PTB (< 37 weeks), and stillbirth.

**Results:**

Of the 1967 screened articles, 17 publications met the inclusion criteria (14 cohort studies, 1 case control and 2 cross-sectional studies). Studies included data from Denmark, UK, Ireland, Nepal, Italy, Israel, Botswana, Australia, China, Netherlands, Saudi Arabia, Austria, Zimbabwe, India, and Spain. The total sample size ranged from 3399 to 1,599,547 pregnant women. Thirteen studies examined PTB with conflicting results, reporting both an increase and a decrease in PTB incidence, with odds ratios [95% CI] ranging from 0.09 [0.01, 0.40] to 1.93 [0.76, 4.79]. Three studies found a decrease in LBW rates during lockdowns, one of which was statistically significant, with a rate ratio of 3.77 [1.21, 11.75]. Ten studies examined stillbirth rates, including four studies reporting a statistically significant increase in stillbirth rates, with adjusted relative risk ranging from 1.46 [1.13, 1.89] to 3.9 [1.83, 12.0]. Fourteen studies contained data that could be combined in a meta-analysis comparing perinatal outcomes before and during lockdown. We found that lockdown measures were associated with a significant risk of stillbirth with RR = 1.33 [95% CI 1.04, 1.69] when compared to before lockdown period. However, lockdown measures were not associated with a significant risk of PTB, LBW and VLBW compared to prepandemic periods.

**Conclusions:**

This review provides clues about the severity of the indirect influence of COVID-19 lockdown implementation; however, the criteria that lead to unexpected changes in LBW, PTB, and stillbirth remains unclear. Large studies showed conflicting results, reporting both increases and decreases in selected perinatal outcomes. Pooled results show a significant association between lockdown measures and stillbirth rates, but not low birth weight rates. Further studies examining the differences in other countries’ lockdowns and sociodemographic groups from low to middle-income countries are needed. Exploration of perinatal outcomes during COVID-19 lockdown poses an opportunity to learn from and make changes to promote the reduction of the leading causes of childhood mortality worldwide.

**Supplementary Information:**

The online version contains supplementary material available at 10.1186/s12884-021-04156-y.

## Background

COVID-19 has spread worldwide since the World Health Organization (WHO) declared it as a Public Health Emergency of International Concern on 30 January 2020 [1]. In addition to various public health measures, lockdown strategies - of various degrees - were declared in most countries to control the spread of COVID-19. The implementation of lockdowns and the degree of outbreaks had the potential to affect individuals’ health and access to services, like health care, financial benefits, and social support. Although pregnant patients were encouraged not to put their health on hold, as antenatal care is beneficial for maternal and fetal health, there are currently limited clinical reports on the influence of national lockdowns on pregnancy and perinatal outcomes [2].

Globally, the incidence of preterm births (PTB) (10.6%), infants born with a low birth weight (LBW)(14.6%), and stillbirths (1.84%) are declining [3–5]. Although these outcomes’ etiology is not clear, they are associated with factors including environmental conditions and are still most prevalent in low to middle income countries [3–5]. Recently, the media have covered several reports and anecdotes of positive influence of COVID-19, in the form of reduced rates of preterm births observed in hospitals. Several Canadian cities have reported a decrease in preterm births, including a 37% decline in PTB in Calgary, 30% decline in Ottawa, and 80% decline in Halifax [6–8]. Further exploration of perinatal outcome changes during the first wave period poses an opportunity to learn from and make policy changes to promote the reduction of the leading causes of childhood mortality worldwide in the next wave and other pandemics. The objective of this rapid review was to evaluate the impact of COVID-19 lockdowns on the incidence of low birth weight (LBW), preterm birth (PTB), and stillbirth in pregnant women.

## Methods

Two reviewers searched EMBASE, CORD-19, LitCovid (PubMed), WHO Global research on corona virus disease (COVID-19) and MedRxivfor clinical studies published in English from 01 January 2020 to 17 July 2021 using a search strategy comprised of the following terms: “perinatal outcomes” OR “stillbirth” OR “low birth weight” OR “preterm” AND “pregnancy” AND “quarantine” OR “lockdown” AND “COVID-19” and MeSH terms “Pregnancy”, “Pregnancy Outcome”, “Infant”, “Low Birth Weight”, “Stillbirth”, “Birth weight”, “Social Isolation” or “Pandemics”, “Quarantine”, “Premature Birth” or “Infant”, “Premature” and “Coronavirus.” 1967 studies were assessed independently by the screeners (Christine Vaccaro and Farida Mahmoud) using a screening tool (Additional file 1), and any disagreements were resolved through discussion. Studies and accepted articles that reported neonatal and perinatal outcomes of COVID-19 in pregnancy, namely birth weight, preterm birth rate and/or stillbirth rate were included. Furthermore, studies that included singleton or multiple pregnancies were included; however, case reports, review articles, and abstracts presented at international conferences were excluded. The following data were collected from each study: study design, year, country, study period, data source, and whether the study population included singleton or multiple pregnancies. Definition of low birth weight and premature birth, defined by authors in each study, and the estimated Odds Ratio (OR) or Risk Ratio (RR) were extracted, as shown in Tables 1 and 2. The percentage of increase/decrease for each perinatal outcome was calculated when data were available. Data were synthesized using Review Manager for Windows (RevMan, version 5.3, Copenhagen: The Nordic Cochrane Centre, The Cochrane Collaboration, 2014). Risk ratio (RR) was used as the outcome measure. We used the DerSimonian-Laird random-effects model to exclude between-study heterogeneity. Statistical heterogeneity was assessed by the I^2^ statistic and Cochrane’s Q-statistic; *p* <  0.05 indicating significant heterogeneity.

**Table 1:** Studies examining the association between COVID-19 lockdown and premature births and low birth weight rates

Study Ref.	Design	Country	Data Source	Study Period	Pregnancy type	Before Lockdown Period			During Lockdown Period			Effect		
						Definition	Rate/ 1000 live births	***p***-value and/or (95% CI)	Definition	Rate/ 1000 live births	***p***-value and/or (95% CI)	OR or RR	***p***-value and/or (95% CI)	% Decrease/Increase
***Cohort studies***														
Ashish et al. (2020)	Cohort	Nepal	SUSTAIN^1^and REFINE^1^ studies	Jan 1-May 30, 2020	Singletons	< 37 weeks^1^	167	< 0.0001 (0.160, 0.174)	< 37 weeks	200	< 0.0001 (0.178, 0204)	aRR = 1.3	0.0016 (1.2, 1.4)	16.5% increase
						< 2500 g^1^	111	N/A	< 2500 g	105	N/A	N/A	0.37	0.1% NS decrease
Been (2020) [9]	Quasi experimental	The Netherlands	Praeventis (RIVM)	Oct 9, 2010-Jul 16, 2020	Singletons	< 37 weeks +/− 2 months from Mar 9^1^	N/R	N/R	< 37 weeks +/− 2 months from Mar 9	N/R	N/R	OR = 0.77	0.002 (0.66, 0.91)	Decrease
						< 37 weeks +/− 3 months from Mar 9^1^	N/R	N/R	< 37 weeks +/− 3 months from Mar 9	N/R	N/R	OR = 0.85	0.028 (0.73, 0.98)	Decrease
						< 37 weeks +/− 4 months from Mar 9^1^	N/R	N/R	< 37 weeks +/− 4 months from Mar 9	N/R	N/R	OR = 0.84	0.023 (0.73, 0.97)	Decrease
						< 37 weeks +/− 4 months from Mar 15	N/R	N/R	< 37 weeks +/− 4 months from Mar 15	N/R	N/R	OR = 0.96	(0.83–1.10)	NS decrease
						< 37 weeks +/− 4 months from Mar 23	N/R	N/R	< 37 weeks +/− 4 months from Mar 23	N/R	N/R	OR = 1.03	(0.90–1.18)	NSincrease
Hedermann et al. (2020)	Cohort	Denmark	National Screening Biobank (DNSB)	Mar 12-Apr 14, 2015–2020	Singletons	< 28 weeks	2.19	0.003	< 28 weeks	0.19	< 0.001	OR = 0.09	< 0.001 (0.01, 0.40)	90.0% decrease
						28–31 weeks	5.57	N/A	28–31 weeks	6.62	N/A	OR = 1.11	0.589 (0.75, 1.61)	15.9% NS increase
						32–36 weeks	42.8	N/A	32–36 weeks	41.84	N/A	OR = 0.98	0.742 (0.84, 1.13)	2% NS decrease
Khalil et al. (2020) [10]	Cohort	United Kingdom	St. George’s University Hospital	Oct 1-Jun 21, 2019–2020	Singletons, Twins & Triplets	< 37 weeks	68	N/A	< 37 weeks	76	N/A	cOR = 1.11	0.46 (−2.43, 1.07)	10.5% NS increase
						< 34 weeks	25	N/A	< 34 weeks	37	N/A	cOR = 1.46	0.07 (−0.05, 2.30)	32.4% NS increase
Philip et al. (2020) [11]	Cohort	Ireland	UMHL^1^ VON^1^ CSO^1^	Jan 1-Apr 31, 2001–2020	Singletons & Multiple births	< 1500 g^1^	8.18	(7.21, 9.29)	< 1500 g	2.17	0.022 (1.21, 11.75)	Rate ratio = 3.77	0.022 (1.21, 11.75)	73.5% decrease
						< 1000 g^1^	2.17	(2.43, 3.70)	< 1000 g	0	N/A	N/A	N/A	100% NS decrease
Matheson et al. (2021) [12, 13]	Cohort	Australia	3 maternity hospitals	July-Sep 2019–2020	Singleton and multiple	< 37 weeks	101	N/A	< 37 weeks	83	N/A	OR = 0.81	(0.67–0.98)	Decrease
						< 34 weeks	36	N/A	< 34 weeks	26	N/A	OR = 0.71	(0.51–0.98)	Decrease
						< 28 weeks	8	N/A	< 28 weeks	4	N/A	OR = 0.46	(0.21–0.99)	Decrease
De Curtis et al. (2020) [14]	Cohort	Italy	Lazio (nationwide)	Mar-May 2019–2020	Singletons	< 32 weeks	5.5	N/A	< 32 weeks	7.9	N/A	N/A	0.06	NS increase
						32–36 weeks	59.3	N/A	32–36 weeks	46.2	N/A	N/A	< 0.001	Decrease
Justman et al. (2020) [15]	Cross sectional	Israel	Tertiary center	Mar-Apr 2019–2020	Singleton and multiple	< 37 weeks	65	N/A	< 37 weeks	64	N/A	N/A	0.96	NS decrease
Caniglia et al. (2021) [16]	Cohort	Botswana	Tsepamo study	Jan1-July20 2019–2020	Singletons	< 32 weeks	−0.21%	(−0.67, 0.25%)	< 32 weeks	−0.47% (% reduction)	(−1.15, 0.21%)	−0.26% (% reduction)	(−0.80, 0.27%)	NS decrease
						< 37 weeks	0.25%	(−0.66%,1.15%)	< 37 weeks	−1.27% (% reduction)	−2.71%,0.1%)	−1.52% (% reduction)	(−3.14, 0.10%)	NS decrease
Arnaez et al. (2021) [17]	Retrospective	Spain	13 hospitals of the Castilla-y-Léon region	Jan 1-June 21, 2015–2020	Singletons	32–36 weeks	57.4	(5.56–5.92)	32–36 weeks	56.6	(4.83–6.48)	N/A	N/A	NS decrease
						28–31 weeks	6	(0.54–0.66)	28–31 weeks	4.7	(0.22–0.71)	N/A	N/A	NS decrease
						23–27 weeks	2.8	(0.24–0.32)	23–27 weeks	3.7	(0.15–0.58)	OR = 1.38	0.0438(0.61–3.12)	Increase
						< 1500 g	9.8	(0.91–0.105)	< 1500 g	9.8	(0.63–1.33)	N/A	N/A	NS change
						< 1000 g	3.4	(0.29–0.38)	< 1000 g	4.6	(0.22–0.70)	OR = 1.19	0.724(0.44–3.23)	NS increase
Huseynova et al. (2021) [18]	Cross sectional	Saudi Arabia	Public Sector Children Hospital - King Saud Medical City (KSMC)	Mar 1- June 30, 2017–2020	Singleton and multiple	< 28 weeks	19.87	N/A	< 28 weeks	13	N/A	RR = 63.8%	0.047	36.2% decrease
						28–31 weeks	29.13	N/A	28–31 weeks	31.2	N/A	RR = 101.20%	0.398	NS increase
						32–36 weeks	144.13	N/A	32–36 weeks	106.6	N/A	RR = 73.66%	0.0004	26.34% decrease
Kirchengast et al. (2021) [19]	Retrospective medical record-based single-centre	Austria	Viennese Danube Hospital and Statistics Austria	Jan 1- July 31, 2017–2020	Singleton	< 1000 g	7.2	N/A	< 1000 g	2.9	N/A	OR = 2.48	0.35–17.50	Decrease
						< 1500 g	18	N/A	< 1500 g	8.9	N/A	OR = 2.05	0.63–6.67	Decrease
						1500-2500 g	54.2	N/A	1500-2500 g	38.9	N/A	OR = 1.41	0.76–2.62	Decrease
						< 32 weeks	28.9	N/A	< 32 weeks	14.9	N/A	OR = 1.93	(0.76–4.79)	Decrease
						32–36 weeks	54.2	N/A	32–36 weeks	62.9	N/A	OR = 1.01	(0.97–1.05)	Increase
Gallo et al. (2021)	Retrospective	Australia	Mater Mothers’ electronic healthcare records database	March 16–May 1, 2013–2020	Singletons	23–27 weeks	6.70	N/A	23–27 weeks	8.35	N/A	aOR = 1.21	(0.69–4.20)	NS increase
						28–31 weeks	10.46	N/A	28–31 weeks	5.97	N/A	aOR = 0.55	(0.22–1.75)	NS decrease
						32–26 weeks	59.51	N/A	32–26 weeks	34.61	N/A	aOR = 0.57	(0.35–0.80)	Decrease

^1^ISRCTN18148368

**Table 2:** Studies examining the association between COVID-19 lockdown and the rates of stillbirth

Study Ref.	Design	Country	Data Source	Study Period	Pregnancy type	Before Lockdown Period			During Lockdown Period			Effect		
						Definition	Rate/ 1000 total births	***p***-value and/or (95% CI)	Definition	Rate/ 1000 total births	***p***-value and/or (95% CI)	OR or RR	***p***-value and/or (95% CI)	% Reduction/Increase
***Cohort studies***														
Ashish et al. (2020)	Cohort	Nepal	SUSTAIN^1^and REFINE^2^ studies	Jan 1-May 30, 2020	Singletons	Stillbirth^3^	14	< 0.0001 (12, 16)	Stillbirth	21	< 0.0001 (18, 25)	aRR = 1.46	0.0042 (1.13, 1.89)	Increase
De Curtis et al. (2020) [14]	Retrospective	Italy	Lazio hospital discharge database	March–May, 2020	Singletons	Stillbirth	10	N/A	Stillbirth	26	N/A	N/A	0.0017	Increase
Gallo et al. (2021)	Retrospective	Australia	Mater Mothers’ electronic healthcare records database	March 16–May 1, 2013–2020	Singletons	Stillbirth^4^	4.57	N/A	Stillbirth	5	N/A	N/A	0.7	NS change
Khalil et al. (2020) [10]	Cohort	United Kingdom	St. George’s University Hospital	Oct 1-Jun 21, 2019–2020	Singletons, Twins & Triplets	Stillbirth	2.38	N/A	Stillbirth	9.31	N/A	N/A	0.01 (1.83, 12.0)	Increase
Kumari et al. (2020) [20]	Retrospective	India	Tertiary Care Medical College in Western India	Jan 15- Jun 2, 2020	N/A	Stillbirth	22.5	N/A	Stillbirth	31.5	N/A	N/A	0.02	0.9% Increase
Meyer et al. (2020)	Retrospective	Israel	Sheba Medical Center	March 20 – June 27, 2011–2020	Singletons	Stillbirth	10	N/A	Stillbirth	8	N/A	N/A	0.424	NS change
Stowe et al. (2020)	Cohort	United Kingdom	Hospital Episode Statistics	Apr 1 – June 30, 2019–2020	N/A	Stillbirth^5^	4	(0.37–0.44)	Stillbirth	4.1	0.38–0.45	Rate ratio = 1.02	0.69 (0.91–1.15)	NS change
Arnaez et al. (2021) [17]	Retrospective	Spain	13 hospitals of the Castilla-y-Léon region	Jan 1-June 21, 2015–2020	Singletons	Stillbirth	4.4	(0.22–0.7)	Stillbirth	4.6	(0.37–2.18)	OR = 0.90	(0.53–1.79)	NS change
Shakespeare et al. (2021) [21]	Retrospective single-centre cross sectional	Zimbabwe	Mpilo Central Hospital	Jan-June 2020	N/A	Stillbirth	33.1	N/A	Stillbirth	30.09	N/A	N/A	0.81	NS change
Kumar et al. (2021) [22]	Case-control	India	Tertiary Hospital in India	Mar-Sept, 2019–2020	N/A	Stillbirth	29.9	N/A	Stillbirth	37.4	N/A	N/A	0.045	Increase

Abbreviations: *OR* Odds Ratio, *RR* Risk Ratio, *NS* Not Significant, *N/A* Not Associated, *NR* Not Recorded

^1^ISRCTN18148368

^2^ISRCTN16741720

^3^22 weeks, born with no signs of life

^4^19–43 weeks, born with no signs of life

^5^ > 24 weeks, born with no signs of life

## Results

### Preterm birth rates

Since the emergence of COVID-19, eight studies have reported a decrease in national preterm birthrates (Table 1) [9, 12, 14, 16, 18, 19, 23, 24].

The most extensive quasi-experimental study to date (*n* = 1,599,547 singleton newborns) reported a 15–23% decrease in preterm birth rates [9]. Using a regression discontinuity design, Been et al. examined the Netherlands COVID-19 mitigation measures’ impact over several periods of the first wave of COVID-19. Although preterm birth rates decreased across all gestational age categories less than 37 weeks, only the 32–36-week stratum was statistically significant [9].

All three notable dates in the Netherlands COVID-19 mitigation strategy (March 9, March 15, and March23) were further stratified into four-time windows (±1 to ±4 months) [9]. All March 9 windows saw a statistically significant decrease in preterm births (±1 month OR 0.91 [95% CI 0.89, 1.20]; ±2 months OR 0.77 [95% CI 0.66, 0.91]; ±3 months OR 0.85 [95% CI 0.73, 0.98]; ±4 months OR 0.84 [95% CI 0.73, 0.97]).The 15 March 2020 windows saw no significant decreases in preterm births, and after 23 March 2020,there were no statistically significant changes observed [9].

Denmark’s first wave national lockdown began 12 March 2020, with a slow reopening commencing on14 April 2020 [23]. Hedermann et al. looked at the premature birth rates of 31,180 live singleton infants born between 12 March – 14 April from 2015 to 2020. In comparison to previous years, there was an 86.8%significant reduction in infants born less than 28 weeks gestation, OR 0.09 [95% CI 0.01, 0.40] [9]. There were non-significant changes in preterm births for the other two gestational age categories,28–31 weeks (OR 1.11 [95% CI 0.75, 1.61] and 32–36 weeks (OR 0.98 [95% CI 0.84,1.13] [23] (Table 1).

Based on observational birth outcomes surveillance study at 8 governmental maternity wards in Botswana, Caniglia et al., with 68,448 women, examined preterm birth (< 37 weeks) and very preterm birth (< 32 weeks) in singleton newborn recorded before lockdown (January 1, 2020 to April 2, 2020), during lockdown (April 3, 2020 to May 7, 2020), and post-lockdown (May 8, 2020 to July 20, 2020) [16].Using difference-in-differences analysis, 9% relative reduction in preterm birth was associated with the lockdown period (− 1.52% [95% CI, − 3.14 to 0.10%]) compared to pre-lockdown period and decreased by 0.91% (95% CI,-2.57 to 0.75%) during post-lockdown versus pre-lockdown [16]. While for severe preterm birth, a significant decrease by − 0.88% (95% CI, − 1.46% to − 0.31%) during the post-lockdown compared to before lockdown and by − 0.26% (95% CI, − 0.80 to 0.27%) during lockdown versus before lockdown was observed [16]. The greatest impact on the outcomes was shown to be among pregnant women with human immunodeficiency virus and those living in urban areas.

Matheson et al. conducted an interrupted time-series analysis on the monthly rate of preterm birth on singleton and multiple pregnancies in three maternity hospitals in Melbourne during lockdown [13]. The researchers analyzed 2448 births during lockdown (July to September 2020) and 2514 births during the same period in 2019. Significant lower rates of preterm birth were observed before 28 weeks of gestation with OR = 0.45 (95% CI, 0.21–0.99), before 34 weeks (OR 0.71, 95% CI 0.51–0.98), and before 37 weeks (OR 0.81,95% CI, 0.67–0.98) [13]. Matheson et al. found that the effect was independent of multiple pregnancies for births less than 34 weeks with adjusted OR of 0.71 (95% CI, 0.53–0.96) [13].

A retrospective cohort study in Lezio hospital, Italy, by De Curtis et al. examined very preterm birth (< 32 weeks) and late preterm birth rates (32–36 weeks) in singleton births during lockdown period (March to May 2020) and the same period in 2019. There was a non-significant increase (*p* = 0.06) in the rate of very preterm birth 0.55% (*n* = 50) and 0.79% (*n* = 61) before and during the lockdown [14]. Compared with before the lockdown period, the percentage of late preterm births detected during the lockdown period has dropped significantly from 5.93 to 4.62% (*p* <  0.001) [14].

At Sheba Medical Center, Israel, Meyer et al. examined 31,428 singleton pregnancies during 3 periods: lockdown from March 20 to June 27, 2020, the same period during 2019 and a matched period from 2011 to 2019 [24]. Preterm birth rate at less than 34 weeks of gestation was significantly lower in the lockdown period than in both the parallel period in 2019 and matched period from 2011 to 2019with OR = 0.45 [95% CI, 0.30,0.68] and OR = 0.60 [95% CI, 0.41,0.85], respectively [24]. Furthermore, preterm birth at less than 32 weeks of gestation was significantly reduced in the pandemic period compared with the 2019 and 2011–2019 periods; OR = 0.47 [95% CI, 0.27,0.79] and OR = 0.58 [95% CI, 0.37,0.92], respectively [24].

Based on cross-sectional study at the Neonatal Intensive Care Unit, King Saud Medical City, in Saudi Arabia, Huseynova R. et al., with 7226 live births, examined extremely preterm (24–27 weeks + 6 days), very preterm (28–31 weeks + 6 days), and moderate to late preterm (32–36 weeks + 6 days) recorded between March 1 till June 30, 2017–2020 [18]. Among 1320 preterm infants, the authors observed a 23% decline in the overall preterm birth during lockdown period (March1–June 30, 2020) with a signicant 36% (*p* = 0.047) and 26.34% (*p* = 0.0004) prevented fraction of extremely preterm and moderate to late premature births, respectively [18].

Kirchengast S. examined 669 singleton live births in Austria during the lockdown period between March and July 2020 compared to prelockdown period (January–February 2020) and during the last 15 years (2005–2019) [19]. The results showed that the rate of very preterm birth (< 32 weeks) during the lockdown months was markedly lower than prelockdown. However, no significant decrease in late preterm birth rate was observed (OR 1.01, CI 0.97–1.05) [19].

In contrast, some evidence suggests that lockdown had no significant impact on preterm birth rate [10, 15, 17]. A single center cohort study (*n* = 3462 births) by Khalil et al. was conducted at St. George’s University Hospital in the United Kingdom. The investigators observed non-significant increases in preterm births for gestations less than 34 weeks [95% CI -2.43, 1.07] and 37 weeks [95% CI -0.05, 2.30]) (Table 1) [10].

In a large tertiary care center in Israel, Justman et al. conducted a cross-sectional study to examine preterm birth [15]. The researchers compared 310 births during lockdown period (March to April 2020) with 742 births before the pandemic (March to April 2019). A non-significant change was detected in preterm birth at less than 32 weeks (*p* = 0.63) and preterm births at less than 37 weeks (*p* = 0.96) between lockdown period and the same period in 2019 [15].

Arnaez et al. examined 70,024 births born from January 1, 2015, to June 21, 2020, in a population prevalence proportion study across 13 hospitals [17]. Preterm birth rates did not significantly decrease in during the lockdown period or de-escalation period compared to the prelockdown period (OR 0.93, 95% CI 0.75–1.15 and OR 0.99, 95% CI 0.85–1.15, respectively [17].

In Nepal, a national lockdown due to COVID-19 began on 21 March 2020 [25]. A prospective cohort study, with 20,354 mothers by Ashish et al., examined singleton preterm birth rates 2.5 weeks before lockdown (1 January 2020–20 March 2020) compared to 9.5 weeks of lockdown (21 March 2020–30 May 2020). Preterm birth rates (< 37 weeks) increased by 16.5% (aRR 1.3 and 95% CI [1.2, 1.4]) during the lockdown period [25].

**Fig. 1:**
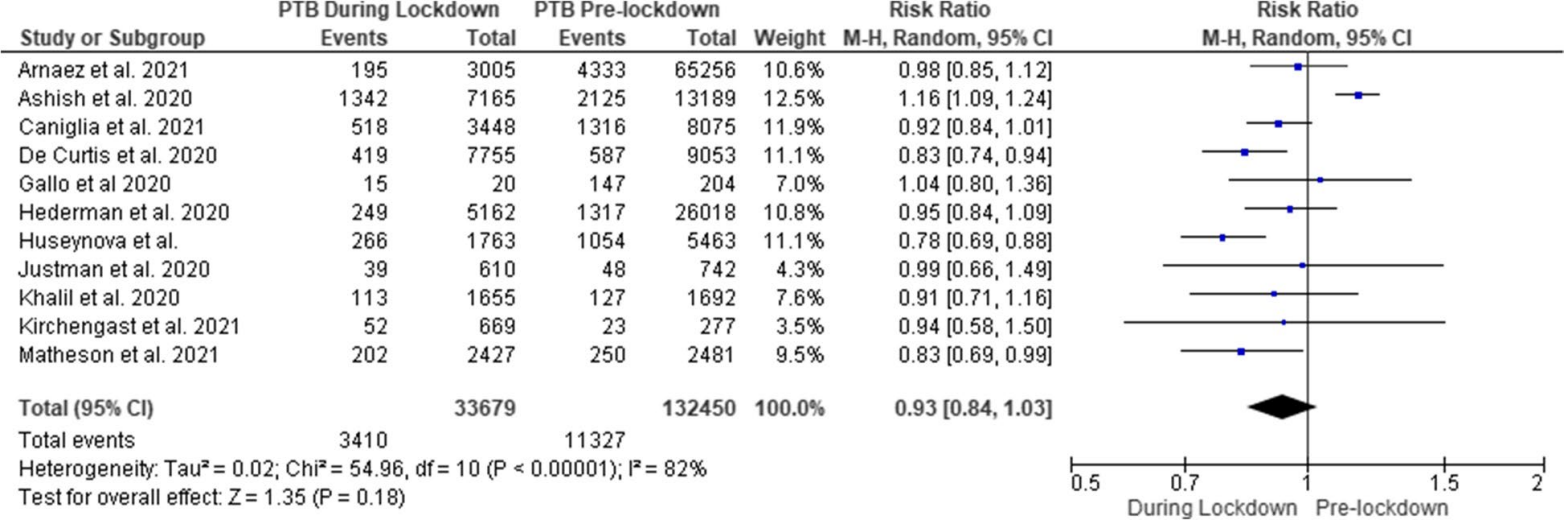
Forest plot of preterm births (< 37 weeks) before and during COVID-19 lockdown periods. *Been et al. 2020 was excluded from the analysis because of lack of preterm birth raw data

In the meta-analysis, there were 3410 PTB (< 37 weeks) among 33,679 singleton and multiple births in the lockdown period compared with 11,327 PTB among 132,450 neonates in the pre-lockdown period. Significant heterogeneity was detected (I^2^ = 82% and *p* <  0.00001) (Fig. 1). Meta-analyses showed that lockdown was not associated with a significant increased/decreased risk of PTB when compared to before lockdown period (RR = 0.93 [95% CI 0.84, 1.03]).

### Low birth weight

Four national cohort studies compared the rates of low birth weight before versus during the first wave of COVID-19 pandemic (Table 1) [11, 17, 19, 25]. The study by Ashish et al. in Nepal on singleton births (*n* = 20,354) reported a non-significant 0.05% decrease (*p* = 0.37) in low birth weight (< 2500 g) rates from before the lockdown (1 January 2020–20 March 2020) to during the lockdown (21 March 2020–30 May 2020) [25]. Philip RK et al. study was entirely dedicated to regional low birth weight trends amongst preterm birth (*n* = 93,018) over two decades, including the period of COVID-19 first wave [11]. Preterm birth starting from 22 weeks of gestation involving low birth weight were divided into two categories: very low birth weight (VLBW) (< 1500 g) and extremely low birth weight (ELBW) (< 1000 g). A 73.5% reduction in VLBW was reported, with a rate ratio of 3.77 [95% CI 1.21, 11.75]. No ELBW live births were recorded during the January–April 2020 period [11].

Kirchengast S. examined 669 singleton live births at Viennese Danube Hospital, Austria during the January–February 2020 and 2005–2019. The results showed that the rate of ELBW (< 1000 g), VLBW (< 1500 g), LBW (1500-2500 g) during the lockdown months (March–July 2020) was lower than prelockdown. The pre-lockdown rate of LBW newborns was significantly higher than the lockdown period (OR 1.66 [95% CI 0.98, 2.81]) [19].

A population prevalence proportion study by Arnaez et al. investigated the rates of LBW singletons in Spain. The authors observed a non significant change in VLBW singletons (< 1500 g). Although the rates of ELBW (< 1000 g) singletons increased from 3.4 to 4.6 per 1000 during the lockdown period (March–May 2020), this finding was not significant (OR 1.19 [95% CI 0.44,2.23]) [17].

**Fig. 2:**
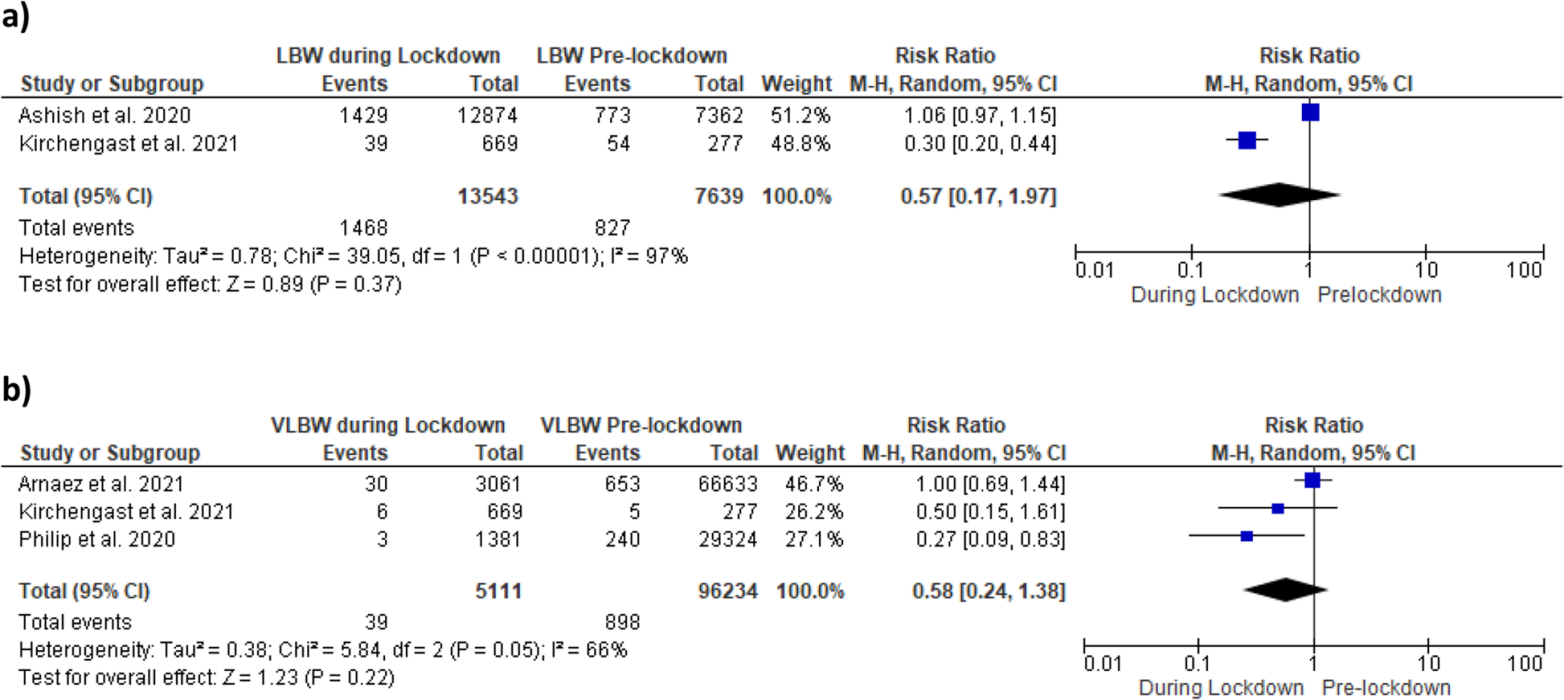
Forest plots of **a** low birthweight and **b** very low birthweight before and during COVID-19 lockdown periods

Meta-analyses showed no significant risk of LBW and VLBW between groups with RR = 0.57 [95% CI 0.24, 1.38 and RR = 0.58 [95% CI 0.17, 1.97], respectively (Fig. 2a, b).

### Stillbirth

Studies have reported that stillbirths across different countries increased [10, 14, 20, 22, 25] or remained unchanged [17, 21, 24, 26, 27] during COVID-19 first wave lockdown. Stillbirth is defined as either fetal death with a gestational age of at least 19 weeks, at least 22 weeks [25] or at least 24 weeks [9, 27] (Table 2). The prospective cohort study in Nepal by Ashish et al. described singleton stillbirth rates (*n* = 20,354) 12.5 weeks before lockdown (1 January 2020to 20 March 2020) and 9.5 weeks during lockdown (21 March 2020–30 May 2020) [25]. Babies born after at least 22 weeks with no signs of life were defined as stillbirths. The adjusted risk ratio of institutional stillbirth rates during the COVID-19 first wave lockdown compared to the period before lockdown was 1.46 [95% CI 1.13, 1.89]) [25].

The previously described cohort study in the United Kingdom by Khalil et al. compared stillbirth rates between the pre-pandemic period (1 October 2019–1 January 2020, *n* = 1681) and the first wave pandemic period (1 February 2020–14 June 2020, *n* = 1718) [10]. Fetal deaths with a gestational age of at least 24 weeks were considered stillbirths. Stillbirth rate during the COVID-19 first wave pandemic period was higher than during the pre-pandemic period, with a difference of 6.93/1000 births [95% CI 1.83, 12.0] [10].

Of the 6209 pregnant women during lockdown, a retrospective analysis by Kumari et al. across four hospitals in India showed that lockdown has resulted in a significant 0.9% increase in stillbirth (*p* = 0.02) compared with the pre-lockdown period [20].

Similarly, a retrospective study in Italy by De Curtis et al. compared the singleton stillbirth rates during the three-month lockdown (March to May) in 2020 to the stillbirth rates during the same months in 2019. A threefold increase in stillbirths was observed when the stillbirth rate went from 10 to 26 per 1000 total births between 2019 and 2020 (*p* = 0.0017) [14].

A case-control study by Kumar et al. compared the rates of stillbirth from March–September 2019 to the same months in 2020 in a single tertiary care center in India. A modest increase, from 29.9 to 37.4 per 1000 births was seen between 2019 and 2020 (*p* = − 0.045) [17].

However, other studies found no significant differences in stillbirth rates before and during the pandemic [17, 21, 24, 26, 27]. In England, Stowe et al. found that the incidence rate ratio of stillbirths during lockdown (April 1, 2020, and June 30, 2020) was 1.02 [95% CI,0.91–1.15]; *p* = 0.69 compared with stillbirth during the same period in 2019. Furthermore, within the 4 English regions, the rate of stillbirth was not significantly different between the pre-pandemic and lockdown periods [27]. Gallo et al. examined stillbirth rates of 10,044 singleton pregnancies at a tertiary perinatal hospital in Queensland from March 16 to May 1, 2020, compared with same period from 2013 to 2019. Stillbirth prevalence did not differ between the examined years (*p* = 0.70) [26]. Meyer et al. collected the singleton stillbirth rates at the Sheba Medical Center, Israel, during the COVID-19 period from March 20 to June 27, 2020 (*n* = 25,940), during a 2019 parallel pre-pandemic period (*n* = 2742), and during parallel annual periods ranging from 2011 to 2019 (*n* = 28,686). It was observed that there was no change between the pandemic and pre-pandemic periods stillbirth rates (*p* = 0.424) [24]. Shakespeare et al. examined stillbirth rates at Mpilo Central Hospital, Zimbabwe from January–March 2020 and April–June 2020 using a cross-sectional design. The rate of stillbirth at Mpilo Central Hospital decreased from 33.1 to 30.09 per 1000 births, however this decline was not statistically significant (*p* = 0.81) [21]. Lastly, a study by Arnaez et al. examined the rate of stillbirth singletons in 13 hospitals located in the Castilla-y-Léon region of Spain. In comparison to the lockdown period, changes in stillbirth rates between the pre-pandemic period in 2020 (OR 0.90 [95% CI 0.37,2.18]) or previous years (OR 1.22 [95% CI 0.45,3.23]) were not significant [17].

**Fig. 3:**
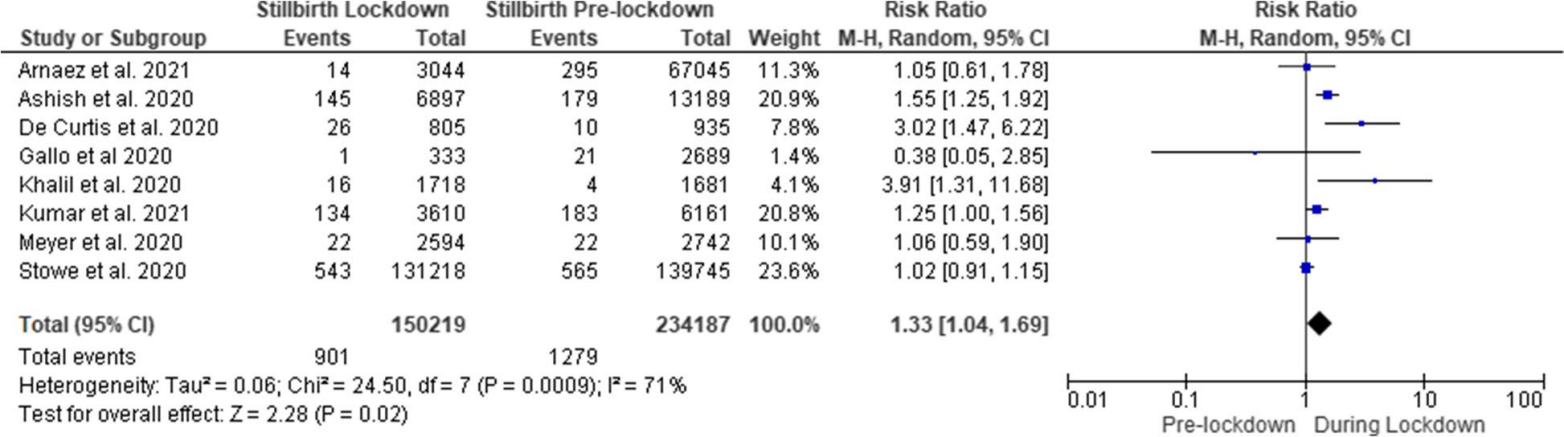
Forest plot of stillbirths before and during COVID-19 lockdown periods. *Kumari et al. 2020 and Shakespeare et al. 2021 were excluded from the analysis because of lack of stillbirth raw data

The meta-analysis included 901 stillbirth among 150,219 neonates in the lockdown period compared with 1279 stillbirth among 234,187 neonates in the pre-lockdown period. Lockdown was associated with a higher risk of still birth than that of pre-lockdown period (RR = 1.33 [95% CI 1.04, 1.69]) (Fig. 3).

## Discussion

The current review described conflicting evidence from large studies reporting both increases and decreases in perinatal outcomes during the COVID-19 first wave lockdowns. The meta-analysis showed a significant association between lockdown measures and increased risk of stillbirths, and no association with preterm births, LBW or VLBW. Preterm birth, low birth weight and stillbirth have puzzled researchers for years to predict and prevent their incidence. However, the socio-environmental, cultural, and economic conditions of the lockdown due to COVID-19 have served to facilitate the understanding of the occurrence of perinatal outcomes during exceptional periods. Studies from Netherlands and Denmark have reported significant reduction in preterm birth rate [9, 11, 23] and non-significant decrease in low birth weight [11, 25]. Additionally, a large study using data from Ireland has adopted very low birth weight and extremely low birth weight infants to evaluate the rate of preterm birth (not the traditional definition of LBW) [11]. Different mechanisms of action were suggested to explain the reported associations. Authors hypothesized that these outcomes are associated with better hygiene measures [9, 23], enhanced public vigilance [16, 28], reduced air pollution [9, 29, 30], increased companionship and social support [26, 31, 32], reduced anxiety [33], less work-related stress [9, 23, 34], and an uptake in maternal wellbeing [9, 29]. However, Been et al. emphasized that in terms of health care and the economy, pandemics and blockades have exacerbated the already existing inequalities among the population [9]. In fact, this is noticeable as underdeveloped countries like Nepal, have rather reported an increase in preterm and still birth rates during the COVID-19 first wave lockdown. Ashish et al. stated that theCOVID-19 first wave has caused women to avoid health facilities and has underlined the overall low-quality of maternal and neonatal care [25]. This highlights that the implementation of lockdowns and the degree of outbreaks had the potential to affect individuals’ health and access to health care. Depending on the disparities faced by these individuals in each study, underreporting of adverse birth outcomes may have occurred. Moreover, mothers in Nepal and Zimbabwe experienced increased stress, due to social restrictions and financial insecurity [25, 32, 35]. Two studies, Been et al. and Gallo et al., described the socioeconomic status of the study population. Gallo et al. adjusted for the socioeconomic status by residence and found no significant difference in extremely preterm or very preterm births between pandemic and prepandemic periods. Additionally, Been et al. tested for effect modification of perinatal outcomes by neighborhood socioeconomic status and it was not statistically significant.

Changes in the adverse outcomes among pregnant women at different pandemic periods suggest that the lockdown could have affected pregnant women to a larger extent. Although two national Cohort studies in Denmark and Botswana reported lower rates of preterm births during the pandemic lockdown, these studies assessed only 1 month of the lockdown period [16, 23]. Canigilia et al. found a greater reduction during the post-lockdown period than during lockdown period in Botswana. This could be attributed to the delayed influence of lockdown restrictions on pregnancy outcomes based on the pregnancy trimester [16]. The analysis captured a diverse set of trial designs to clarify the difference in perinatal outcomes before and during lockdown periods. Lockdown was associated with an increase in stillbirth rate and non-significant change in both PTB, LBW and VLBW when compared with pre-lockdown period.

The current findings have important clinical significance as they provide evidence that first wave lockdown affected perinatal outcomes in different forms. Although Been et al. observed a reduction in preterm births, the authors could not determine if this decline occurred at the expense of high stillbirths’ rates, mainly due to insufficient up-to-date information on stillbirths [9].

Limitations of this rapid review should be acknowledged; the search was limited to articles published in English, the primary research was done within a short period of time, and risk of bias could not be excluded in the included studies. Despite these limitations, this review provides clues about the severity of the indirect influence of COVID-19 first wave lockdown implementation, which appears to be more serious than the direct impact, in the prevention of neonatal outcomes during pandemic. Also, sociodemographic and economic data of low/middle income countries and variation in strict lockdown strategies across countries are worthy of further investigation to develop effective strategies for the second wave and other future pandemics. COVID-19 measures have been implemented with significant variation across countries which resulted in differences in risk factors associated with stillbirth, preterm birth and low birthweight. An international collaborative effort is the essential next step to assess the association between the COVID-19 lockdown measures and still birth rates, which will be crucial for the development of preventive strategies especially in low- to middle-income countries. Further studies are needed to robustly assess whether perinatal complications are affected by COVID-19 lockdown measures compared to pre-pandemic periods. Furthermore, additional research is needed to study major determinants, such as ethnicity and socioeconomic status, and COVID-19 related maternal and neonatal outcomes. With the waves of lockdown currently implemented in several countries, researchers would be able to investigate perinatal outcomes across different demographic strata at different time-periods.

## Conclusions

This review highlights the COVID-19 first wave lockdowns conflicting impact on perinatal outcomes, as evidenced by the recent studies. First wave lockdown was associated with higher risk of reported stillbirth. The observed inconsistent evidence highlights the importance of implementing tailored and rapid-response preventive policies as new evidence emerges. Decision makers should regularly monitor perinatal care and neonatal outcomes throughout the waves of the pandemic, while developing plans for prompt interventions. The results also raise concerns of the social inequalities and healthcare conditions in less developed countries. Nonetheless, the indirect impact of lockdown measures in different countries on perinatal outcomes is worth further study.

### Strengths and limitations of this review

We provided a detailed rapid review and meta-analysis of perinatal outcomes during the COVID-19 first wave lockdowns from developed and developing countries. This review included information on three major perinatal outcomes, stillbirth, preterm births, and low birth weight. As for the limitations of this review, this is a rapid review - not as thorough in nature as a systematic review. 

## Supplementary Information

**Additional file 1.** Screening tool for located studies.

## Data Availability

All materials and data used for this review have been provided in the reference list.

## Abbreviations

COVID-19: Corona Virus Disease 2019
ELBW: Extremely Low Birth Weight
LBW: Low Birth Weight
OR: Odds Ratio
PTB: Preterm Births
RR: Risk Ratio
VLBW: Very Low Birth Weight
