# Supplementary material for: The impact of COVID-19 first wave national lockdowns on perinatal outcomes: a rapid review and meta-analysis
Source: BMC Pregnancy Childbirth. 2021 Oct 6;21:676. doi: 10.1186/s12884-021-04156-y (PMC8532086; doi:10.1186/s12884-021-04156-y)
Supplement: Supplementary file 1 — Additional file 1. Screening tool for located studies. [file 12884_2021_4156_MOESM1_ESM.docx]

**Additional file 1:** Screening tool for located studies

1. Was the article published or made available as a preprint between 1 January 2020 and 17 July 2021?

YES____ NO____ UNCLEAR___

1. Does the study include pregnant women?

YES____ NO____ UNCLEAR____

1. Does the study report at least one outcome of interest (preterm birth (PTB), stillbirth, low birth weight (LBW), or birth weight (BW)?

YES____ NO____ UNCLEAR____

1. Is this a relevant study design (experimental studies, observational studies, etc.)?

YES____ NO____ UNCLEAR____
